# Supplementary material for: Solid Polymer Electrolytes with Flexible Framework of SiO2 Nanofibers for Highly Safe Solid Lithium Batteries
Source: Polymers (Basel). 2020 Jun 10;12(6):1324. doi: 10.3390/polym12061324 (PMC7362020; doi:10.3390/polym12061324)
Supplement: Supplementary file 1 [file polymers-12-01324-s001.pdf]

Supporting information:

Solid polymer electrolytes with flexible framework of SiO_2 nano-fibers for highly safe solid lithium batteries

Jin Cui, Zehao Zhou, Mengyang Jia, Xin Chen, Chuan Shi*, Ning Zhao, Xiangxin Guo*

College of Physics, Qingdao University, 266071 Qingdao, China.

Corresponding author. Tel.: +86 53285950270.

E-mail address: chuanshu@qdu.edu.cn, xxguo@qdu.edu.cn

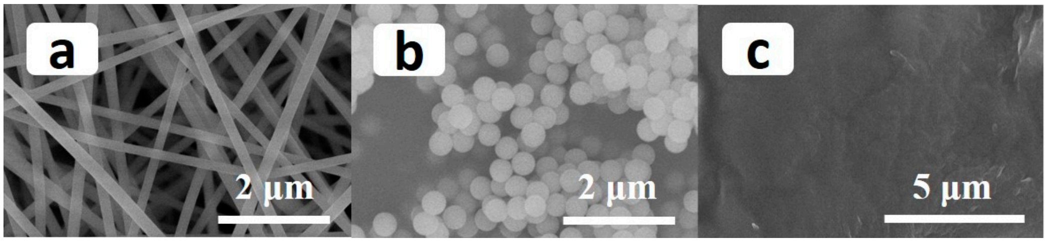

Fig. S1. SEM images of (a) the well-organized framework of 3D SiO_2 NFs, (b) the SiO_2 particles. (c) Top view image of PLS with a smooth surface.

The top-view SEMs of 3D SiO_2 NFs, SiO_2 particles and PLS are shown in Fig. S1. The frameworks of 3D SiO_2 NFs consist of nano-fibers in diameter of approximately 200 nm. In contrast, SiO_2 particles in diameter of approximately 250 nm are prepared, of which SEM images are shown in Fig. S1b.

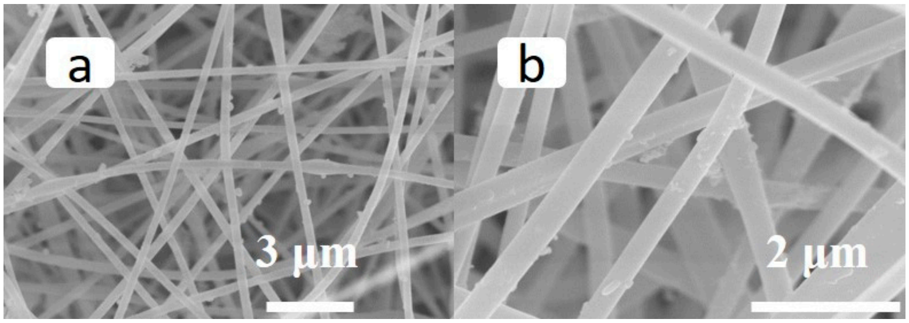

Fig. S2. SEMs of PLS/3D SiO_2 NFs after heated with alcohol burner.

After being heated with alcohol burner, the melted PEO is absent while the structure of 3D SiO_2 NFs maintains. This feature is beneficial to avoiding short circuit at elevated temperatures.
